# Effects of posterior intrusion using skeletal anchorage on treating anterior open bite: A systematic review and meta-analysis

**DOI:** 10.34172/joddd.2023.40754

**Published:** 2023-12-30

**Authors:** Maryam Omidkhoda, Erfan Bardideh, Arezoo Jahanbin, Milad Zarei

**Affiliations:** Department of Orthodontics, School of Dentistry, Mashhad University of Medical Sciences, Mashhad, Iran

**Keywords:** Anterior open bite, Posterior intrusion, Skeletal anchorage

## Abstract

**Background.:**

Posterior intrusion with skeletal anchorage is one of the effective methods in the treatment of anterior open bite. Knowing the effects of posterior intrusion, the amount of possible molar intrusion using skeletal anchorage, and its impact on clinical and cephalometric indicators can help the clinician choose the optimal treatment method, especially in borderline surgical cases.

**Methods.:**

In this systematic review, a series of articles were collected through a systematic search in databases, and the titles and summaries of all these articles were reviewed. After removing the irrelevant articles, the full texts of the related articles were read carefully, and their validity was evaluated. Only RCTs and observational studies that complied with PICO questions were included. The Cochrane Risk of Bias 2.0 (RoB 2), ROBINS-I, and GRADE were used to assess the risk of bias in the included studies. The relevant information on selected articles was extracted, and a meta-analysis was performed with Review Manager 5.4 software.

**Results.:**

The meta-analysis revealed a significant average molar intrusion of 2.89 mm using temporary anchorage devices (TADs). A subgroup analysis showed that miniplates achieved greater intrusion (3.29 mm) compared to miniscrews (2.25 mm) (*P*=0.03). The level of applied force did not significantly affect the degree of intrusion. Dental parameters such as overbite and overjet were notably altered, with overbite increasing by 4.81 mm and overjet decreasing by 2.06 mm on average. As for the skeletal cephalometric characteristics, SNB, ANB, and SN-Pog increased while mandibular plane angle and lower anterior facial height (LAFH) decreased, and these changes were significant. Meanwhile, SNA and palatal angle changes were not significant.

**Conclusion.:**

TADs have proved effective in achieving significant intrusion of maxillary molars, leading to marked improvements in dental and skeletal characteristics in patients with open bite malocclusion. Miniplates proved more effective in achieving greater intrusion.

## Introduction

 Anterior open bite, a challenging malocclusion in orthodontic treatment, is characterized by insufficient vertical overlap between the upper and lower teeth. This multifactorial malocclusion results from various etiological factors, including skeletal, dental, respiratory, neurological, or habitual ones. Addressing these challenges requires a range of treatment options, from habit elimination and dental treatment to surgical intervention.^1,2^

 Elimination of oral habits like finger sucking or pacifier use is critical to prevent dental and skeletal effects at an early age. Depending on the patient’s specific needs, dental treatments, including incisor extrusion, premolar extractions, and posterior impaction, can be implemented to correct anterior open bite. Incisor extrusion is a standard treatment for patients with a normal skeletal pattern or those with vertical dysplasia whose incisors are less visible when resting and smiling.^3^

 Surgical treatments, such as segmental LeFort I osteotomy, offer an alternative for patients who cannot achieve the desired therapeutic results through other methods. It is typically performed after growth completion to reduce vertical maxillary excess and flatten the occlusal plane.^3^

 Posterior impaction using temporary anchorage devices (TADs) has gained popularity recently because it allows for more predictable, effective, and efficient tooth movement. These devices offer infinite anchorage, enabling more precise dental movements. TADs can be placed in the bone transosteally, subperiosteally, or endosteally and mechanically (cortical stabilized) or biomechanically (osteointegration) fixed to the bone. These devices have revolutionized orthodontics by creating a new concept called infinite anchorage (zero anchorage loss).^4^

 Various types of TADs, such as miniplates and mini-screws, are available for posterior impaction. The choice of TAD depends on clinical factors like the amount of intrusion required, the number of teeth that need intrusion, the axial inclination of the teeth, and more. The intrusive force can be applied directly to the teeth or through bite plates, providing versatile options for orthodontic treatment.^5^

 However, despite the various treatment options, studies on the effects of posterior intrusion with skeletal anchorage have reported conflicting results, leading to a debate on the best method, whether miniplate or mini-screw, for posterior impaction using TADs. Investigating the efficacy and outcomes of different TADs in treating anterior open bite is essential in determining the most effective approach for orthodontic practitioners. Therefore, our study investigated the effects of posterior dental intrusion using skeletal anchorage in patients with open bite through a systematic review and meta-analysis.

## Methods

 The present study was a systematic review and meta-analysis based on the PRISMA (Preferred Reporting Items for Systematic Reviews and Meta-Analyses) checklist.^6^ Additionally, the protocol for this meta-analysis was registered on PROSPERO with the code CRD42022308145.

 The PICO framework for this study is outlined as follows:

- P (Population): Adult patients with anterior open bite malocclusion
- I (Intervention): Posterior maxillary intrusion using skeletal anchorage
- C (Comparison): Patients with anterior open bite malocclusion, who received other treatments for anterior open bite, or baseline characteristics of patients who underwent TAD
- (Outcome): Cast measurements and cephalometric indices

###  Inclusion and exclusion criteria

 The inclusion criteria for this two-part study, consisting of a “systematic review” and “meta-analysis,” were:

- Articles examining posterior maxillary intrusion using TADs
- Human sample target groups

The exclusion criteria were:

- Case reports/case series or studies without a TAD group
- Studies on patients with maxillofacial congenital anomalies
- Animal-based research

###  Search strategy and sources

 For the systematic review portion of our study, we first developed a systematic search strategy using keywords relevant to the research question. Using this strategy, we searched PubMed, Scopus, Embase, Google Scholar, Web of Science, and the Cochrane Central Register of Controlled Trials databases from inception to September 12, 2022. All the retrieved articles were thoroughly reviewed. To explore gray literature sources for conference proceedings, we used the terms “mini-screw” and “posterior intrusion.” We also searched the Clinicaltrials.gov and WHO databases for ongoing trial protocols. In addition, we manually searched the references of included studies and relevant studies in high-impact orthodontic journals (IF > 1) from 2004 to 2022. A unique systematic search strategy was used for each database, as shown in Table 1.

**Table 1:** Databases, applied search strategy, and numbers of retrieved studies

**Database of published trials, dissertations and conference proceedings**	**Search strategy used**	**Hits**
MEDLINE searched via PubMed searched on December 22, 2022, via https://www.ncbi.nlm.nih.gov/sites	#1 TAD OR temporary anchorage device OR Mini-screw OR micro-implant OR zygomatic implant OR mini-plate OR titanium plate OR surgical plate OR zygomatic anchorage #2 posterior impaction OR molar intrusion OR molar impaction OR open-bite OR long face OR high-angle mandible #3 #1 AND #2 496	496
Web of science Core Collection was searched via web of knowledge on December 22, 2022, via apps. webofknowledge.com	#1 TS = (TAD OR temporary anchorage device OR Mini-screw OR micro-implant OR zygomatic implant OR mini-plate OR titanium plate OR surgical plate OR zygomatic anchorage) #2 TS = (posterior impaction OR molar intrusion OR molar impaction OR open-bite OR long face OR high-angle mandible) #3 #1 AND #2 267	267
EMBASE searched via Ovid on December 20, 2022, via http://ovidsp.dc2.ovid.com	#1 ((((tad OR 'temporary anchorage device'/exp OR 'temporary anchorage device' OR 'miniscrew'/exp OR 'miniscrew' OR 'mini screw' OR mini) AND ('screw'/exp OR screw) OR 'miniplate'/exp OR 'miniplate' OR 'mini plate' OR mini) AND plate OR surgical) AND plate OR zygomatic) AND anchorage #2 ((posterior AND ('impaction'/exp OR impaction) OR 'molar'/exp OR molar) AND ('intrusion'/exp OR intrusion) OR long) AND ('face'/exp OR face) OR 'long face'/exp OR 'long face' OR 'open bite'/exp OR 'open bite' OR openbite OR 'malocclusion'/exp OR 'malocclusion' #3 #1 AND #2	120
Scopus searched via Scopus on December 21, 2022, via https://www.scopus.com	ALL (tad OR temporary AND anchorage AND device OR mini AND screw OR mini-screw OR micro AND implant OR micro-implant OR zygomatic AND implant OR mini AND plate OR mini-plate OR surgical AND plate OR zygomatic AND anchorage ) AND ALL ( posterior AND impaction OR molar AND impaction OR open AND bite OR open-bite OR long AND face OR long-face OR high AND angle AND mandible )	45
Cochrane Central Register of Controlled Trials searched via the Cochrane Library Searched on December 19, 2022, via https://www.cochranelibrary.com/	#1 TAD 251 #2 anchorage device 80 #3 mini-screw 36 #4 mini screw 104 #5 micro implant 258 #6 mini plate 136 #7 surgical plate 1313 #8 zygomatic anchorage 9 #9 molar impaction 141 #10 molar intrusion 38 #11 posterior intrusion 46 #12 long face 3541 #13 open bite 390 #14 #1 OR #2 OR #3 OR #4 OR #5 OR #6 OR #7 OR #8 2020 #15 #9 OR #10 OR #11 OR #12 OR #13 4074 #16 #14 AND #15 71	21
Total	949	

###  Study selection and data extraction

 The data extraction process was conducted per the Cochrane Handbook for Systematic Reviews.^7^ Two researchers independently reviewed the titles and abstracts of relevant studies. They eliminated studies that failed to meet the inclusion and exclusion criteria. Any disagreements between them were resolved through discussion. Next, they obtained and assessed the full texts of the remaining studies for inclusion in the systematic review and meta-analysis.

 Data from the selected articles were extracted, and the accuracy of the extraction was verified. The information gathered included authors’ names, publication year, study type (randomized, non-randomized, cohort, before-after), patient numbers in treatment and control groups, patients’ mean age, participants’ gender, study duration, inclusion and exclusion criteria, types of TADs used, force applied, number of TADs used, effects on overbite, overjet, and cephalometric indices. The data summary for the relevant studies can be found in Table 2.

**Table 2:** A summary of the characteristics of the included studies

**Author, Year**	**Study design**	**Sample size**	**Gender**	**Sample age**	**Type of anchorage**	**Applied force location**	**Number of miniscrews/miniplates**	**Applying force**	**Force per side**	**Intrusion time**	**Amount of posterior teeth intrusion**	**Assessment**
Abdulnabi,^5^ 2016	Prospective	15	7M, 8F	20.6 ± 4	Miniscrew	Teeth plate	4 (2 in buccal, bw 5,6-6,7)	2 coil springs each side (G&H)	250 g	3-8.2 months (mean 6.3, SD 1.4)	2.9 ± 1.2 mm	OB
de Oliveira,^8^ 2015	Retrospective	9	3M, 6F	18.7 ± 5.1	Miniplate	Teeth	2	Elastic chain	450 g	6 months	2.03 ± 0.87 mm	MP angle- OP angle – PP angle
Kuroda,^9^ 2007	Prospective	23 (10 I, 13 S	0 M, 10 F (I) - 4 M, 9 F (S)	21.6 ± 7.3	Miniplate/miniscrew	Teeth	2	Elastic chain	150 g	-	3.6(I), 0.2(S) ± 1.6(I), 1.5(S) mm	MP angle – SNA – SNB – ANB – U1-FH(A) – L6-MP(D) – L1-MP(D) – U6-PP(D) – OJ – OB – U1-pp(D)
Scheffler,^10^ 2014	Retrospective	30	11M, 19F	24.1 ± 10.7	Miniplate/miniscrew	Bite plate	2	2 coil springs each side	150 g	0.5 ± 0.1 years	2.3 ± 1.4 mm	MP angle – U6-PP(D) - OB
Erverdi,^11^ 2007	Prospective	11	5M,6F	19.5	Miniplate	Bite plate	2	2 coil springs each side	400 g	9 months	3.6 ± 1.4 mm	MP angle – OP angle - SNA – SNB – ANB – U6-PP(D) – OJ - OB
Erverdi,^12^ 2004	Prospective	10	-	17-23	Miniplate	Teeth	2	2 coil springs each side	-	5.1 months	2.6 ± 1.39 mm	MP angle- OP angle – PP angle - SNA – SNB – ANB - U1-PP(D) - L6-MP(D) - L1-MP(D) - U6-PP(D) – OJ - OB
Elshal,^13^ 2021	Prospective	10	4M, 6F	22.4 ± 3.2	Miniplate	Hyrax with bite plate (with 4mm exp)	2	2 coil springs each side	250 g	9.7 ± 2.07 months	3.85 ± 0.82 mm	SNA – SNB – ANB – SNPog - U1-PP(D) - L6-MP(D) - U6-PP(D)
Seres,^14^ 2009	Prospective	7	3M, 4F	21	Miniplate	Teeth	2	1 coil springs each side	100 – 120 g	6 months	-	MP angle
Kim,^15^ 2018	Retrospective	21	3M, 18F	23.9	Miniscrew	Teeth	-	-	-	9.7 ± 3.2 months	2.2 ± 0.8 mm	SNA – SNB – ANB - U1-PP(D) - L6-MP(D) - U6-PP(D) - L1-MP(D) – OJ – OB – MP angle
Turkkahraman, ^16^ 2016	RCT	40(20 I, 20 C)	I: 6M,14F - C: 9M,11F	I: 16.68 ± 2.8 - C: 16.63 ± 2.83	Miniplate	Teeth (hyrax without bite plate)	2	1 coil springs each side	200 g	-	3.59 ± 1.34 mm	MP angle - OP angle – U6-PP(D) – OJ – OB – upper lip E line – lower lip E line
Marzouk,^17^ 2018	Retrospective	28	-	19-28	Miniplate	Teeth	2	1 coil springs each side	450 g	-	3.04 ± 0.79 mm	U6-PP(D) – OJ – OB - upper lip E line – lower lip E line
Akl,^18^ 2020	RCT	20(10I, 10C)	-	18.95 ± 1.77(I) - 19.22 ± 1.45©	Miniscrew		4(2 in buccal bw 6,7 and 2 in palatal)	4 coil springs each side	400(I), 200© g	-	2.26(I), 2.42© ± 1.87(I), 2.06© mm	OB
Marzouk,^19^ 2015	Prospective	13	4M, 9F	18.8 ± 2.2	Miniplate	Teeth	2	2 coil springs each side	450 g	9 ± 2.5 months	3.1 ± 0.74 mm	MP angle - OP angle – PP angle - SNA – SNB – ANB - SNPog - U1-FH(A) – U6-PP(D) - L1-MP(D) – OJ - OB
Akan,^20^ 2020	Prospective	19	5M, 14F	16.5	Miniplate	Hyrax with bite plate	2	3 coil springs each side	400 g	9.4 ± 0.7 months	2.32 ± 2.13 mm	MP angle - OP angle – PP angle - SNA – SNB – ANB - L6-MP(D) - L1-MP(D) – OJ – OB – upper lip E line – lower lip E line
Marzouk,^21^ 2016	Prospective	26	-	-	Miniplate	Teeth	2	2 coil springs each side	450 g	-	3.04 ± 0.79 mm	MP angle – SNPog - SNA – SNB – ANB - U1-FH(A) – U6-PP(D) - L1-MP(D) - L6-MP(D) – OJ - OB
Ari-Demirkaya,^22^ 2005	Retrospective	32 (16I, 16C)	3M, 13F	19.25(I), 19.43©	Miniplate	-	2	2 coil springs each side	-	-	-	Root Resorption
Kassem,^23^ 2018	Prospective	26	11M, 15F	22.4 ± 2.3	Miniplate	Teeth	2	1 coil springs each side	450 g	-	3.04 ± 0.79 mm	MP angle – SNPog - SNA – SNB – ANB - U1-FH(A) – U6-PP(D) - L1-MP(D) - L6-MP(D) – OJ - OB

*M = Male, F = Female, I = Intervention, C = Comparison, S = Surgery.

###  Risk of bias assessment

 To evaluate the risk of bias in randomized clinical trials, the Cochrane Risk of Bias tool for randomized trials (RoB 2) was employed, while the Cochrane’s Risk of Bias in Non-randomized Studies - of Interventions (ROBINS-I) questionnaire was used for non-randomized studies.^24,25^ The RoB 2 questionnaire comprises five domains, with answers categorized as low risk, some concerns, or high risk. Similarly, the ROBINS-I questionnaire has seven domains, with judgments (low, moderate, serious, or critical) based on the degree of bias in the studies.

 Additionally, the quality and confidence of the evidence and meta-analytic results were appraised using the Grading of Recommendations Assessment, Development, and Evaluation ranking system (GRADE). The GRADE system evaluates the quality and reliability of evidence based on several factors, such as the type of articles, risk of bias, risk of non-uniformity of results, indirect evidence, inaccuracy in the results, and other cases.^26^

 The quality and confidence of evidence are classified into four levels: high, moderate, low, and very low. For instance, a low-confidence rating indicates that the meta-analytic result can be extended to clinical conditions with low confidence. Table 3 summarizes the quality assessment of the analysis of landmark data before and after treatment, using the GRADE classification.

**Table 3:** GRADE assessment of certainty and quality of the evidence

**Certainty assessment**							**No of patients**		**Effect**		**Certainty**
**No. of studies**	**Study design**	**Risk of bias**	**Inconsistency**	**Indirectness**	**Imprecision**	**Other considerations**	**Cephalometric skeletal**	**Placebo**	**Relative** **(95% CI)**	**Absolute** **(95% CI)**	
SNA
8	Observational studies	Serious^a^	Not serious	Not serious	Not serious	None	120	120	-	MD 0.1 lower (0.66 lower to 0.46 higher)	⨁⨁⨁◯ Moderate
SNB
8	observational studies	Serious^b^	Not serious	Not serious	Not serious	None	120	120	-	MD 2.07 higher (1.51 higher to 2.64 higher)	⨁⨁⨁◯ Moderate
ANB
8	observational studies	Serious^a^	Serious^b^	Not serious	Not serious	None	120	120	-	MD 1.97 lower (2.95 lower to 0.99 lower)	⨁⨁◯◯ Low
LAFH
8	observational studies	Serious^a^	Serious^b^	Not serious	Not serious	None	139	139	-	MD 2.88 lower (3.89 lower to 1.87 lower)	⨁⨁◯◯ Low
MP Angle
10	observational studies	Serious^b^	Not serious	Not serious	Not serious	None	169	169	-	MD 1.91 lower (2.83 lower to 0.99 lower)	⨁⨁⨁◯ Moderate
U1-PP
4	observational studies	Serious^a^	Not serious	Not serious	Not serious	None	51	51	-	MD 0.62 higher (0.37 lower to 1.61 higher)	⨁⨁⨁◯ Moderate
U6-PP
11	observational studies	Serious^a^	Not serious	Not serious	Not serious	None	190	190	-	MD 2.89 lower (3.34 lower to 2.45 lower)	⨁⨁⨁◯ Moderate
Overbite
12	observational studies	Serious^a^	Serious^b^	Not serious	Not serious	None	214	214	-	MD 4.81 higher (3.87 higher to 5.76 higher)	⨁⨁◯◯ Low
Overjet
9	observational studies	Serious^a^	Not serious	Not serious	Not serious	None	158	158	-	MD 2.06 lower (2.66 lower to 1.45 lower)	⨁⨁⨁◯ Moderate

###  Statistical analysis

 We assessed differences before and after treatment using the mean difference (MD) with a 95% confidence interval (CI) due to the continuous nature of all the examined variables. Due to differences in treatment methods, comparison groups, skeletal anchorage, and examination times, we performed random-effect meta-analyses to investigate the status of cephalometric indices, including SNA, SNB, ANB, S-N-Pog, mandibular plane angle, occlusal plane angle, palatal plane angle, upper lip to E-line, lower lip to E-line, U1-FH (angle), U1-PP (distance), U6-PP (distance), L1-MP (distance), L6-MP (distance), lower anterior facial height (LAFH), IMPA, overjet, and overbite, given their common assessment across studies.

 Data regarding treatment and control groups were extracted from the studies, and the confidence intervals and mean differences were calculated using Review Manager 5.4 software (Copenhagen, Denmark). Information from the selected articles was deemed suitable for meta-analysis if the therapeutic intervention and results were similar.

 We used Cochran’s Q test and the I^2^ test to assess and measure heterogeneity between studies, respectively. I^2^ values < 30% indicate low heterogeneity, values 30‒60% suggest moderate heterogeneity, and values > 60% indicate significant heterogeneity. In cases of high heterogeneity, we attempted to examine study results more homogeneously by conducting subgroup analyses and separating studies with different comparison groups and outlying results.

## Results

 A total of 949 articles were identified through database searches (Medline: 496; Web of Science: 267; Embase: 120; Scopus: 45; Cochrane Central: 21) and hand searching related to the study’s title. After removing 257 duplicates, 674 articles remained, and their titles and abstracts were reviewed. Around 613 articles were subsequently excluded based on the study’s inclusion and exclusion criteria, which comprised 103 animal and case report studies, 171 review articles or book chapters, 131 studies unrelated to TADs, 132 studies without an open bite group, and 76 vertical dimension control studies.

 The full texts of the remaining 61 studies (51 from database searches and 10 from the hand search) were obtained and evaluated. Of these, 11 studies were excluded for only examining vertical dimension control, 10 for having their datasets already included, 17 for being case series, and 6 for being finite element analysis studies. Ultimately, 17 articles were selected for the systematic review.

**Figure 1:**
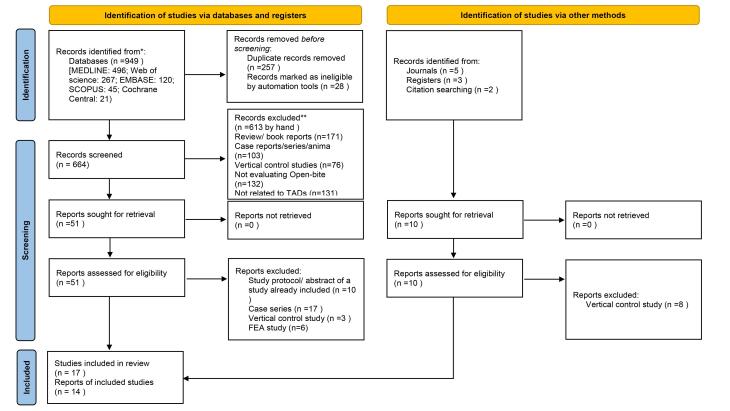
PRISMA study flow diagram

 Upon data extraction, meta-analyses were conducted on 14 studies.^5,9-13,15-22^ For the study by Kassem et al,^23^ only a systematic review was performed due to the same data as the study by Marzouk et al,^21^ and for two studies due to differences in the outcomes studied with other studies.^8,14^ The PRISMA diagram illustrating the selection of relevant studies can be found in Figure 1.

###  Characteristics of Included Studies

 Among all studies, 14 were selected for meta-analysis, which were conducted between 2004 and 2020.^5,9-13,15-22^ Table 2 summarizes the study characteristics. Several studies have investigated the use of miniplates in orthodontic treatment. Some studies used miniplates with posterior bite plates.^5,12,13,20^ These studies applied forces ranging from 250 g to 400 g using coil springs, with intrusion times varying between 3 and 9.7 months. The amount of posterior teeth intrusion achieved in these studies ranged from 2.3 mm to 3.85 mm.

 In contrast, other studies used miniplates with teeth as the anchorage site.^8,14,16,17,19,21^ These studies applied forces using elastic chains or coil springs, with forces ranging from 100 to 450 g. Intrusion times and posterior teeth intrusion amounts varied, with some studies not reporting these values.

 A few studies investigated using both miniplates and miniscrews.^9,10^ Kuroda et al^9^ used elastic chains to apply a force of 150 g, while Scheffler et al^10^ used coil springs with a force of 150 g. Kuroda et al^9^ found varying amounts of intrusion between the two groups, while Scheffler et al^10^ reported a mean intrusion of 2.3 mm.

 Some studies utilized miniscrews as the anchorage method.^5,15,18^ Abdulnabi et al^5^ used bite plates and coil springs to apply a force of 250 g, whereas Akl et al^18^ applied forces of 400 g and 200 g with four coil springs. Kim et al^15^ did not report the method of force application but reported an intrusion time of 9.7 months and an intrusion amount of 2.2 mm. Lastly, Ari-Demirkaya et al^22^ conducted a retrospective study using miniplates without specifying the anchorage site. They utilized two coil springs for force application but did not provide details on force magnitude or intrusion time. Instead, they focused on root resorption as their primary assessment outcome.

###  Risk of bias assessments

 The quality of two randomized clinical trials, Akl et al^18^ and Turkkahraman et al,^16^ was assessed using Cochrane’s Risk of Bias (RoB 2) analysis. Both studies had some concerns regarding the overall risk of bias. Turkkahraman et al^16^ did not clearly state the randomization and concealment methods (randomization process), did not implement blinding of patients and interventionists (deviations from the intended interventions), and did not have registered protocols (selection of the reported result).

 In Akl and colleagues’ study,^18^ the randomization and concealment methods were clearly stated (randomization process), blinding of patients and interventionists was implemented (deviations from the intended interventions), all study patients completed the study (missing outcome data), and the outcome measurement methods were consistent with the study’s objectives (measurement of the outcome). This study was only classified as having some concerns due to the absence of registered protocols.

**Figure 2:**
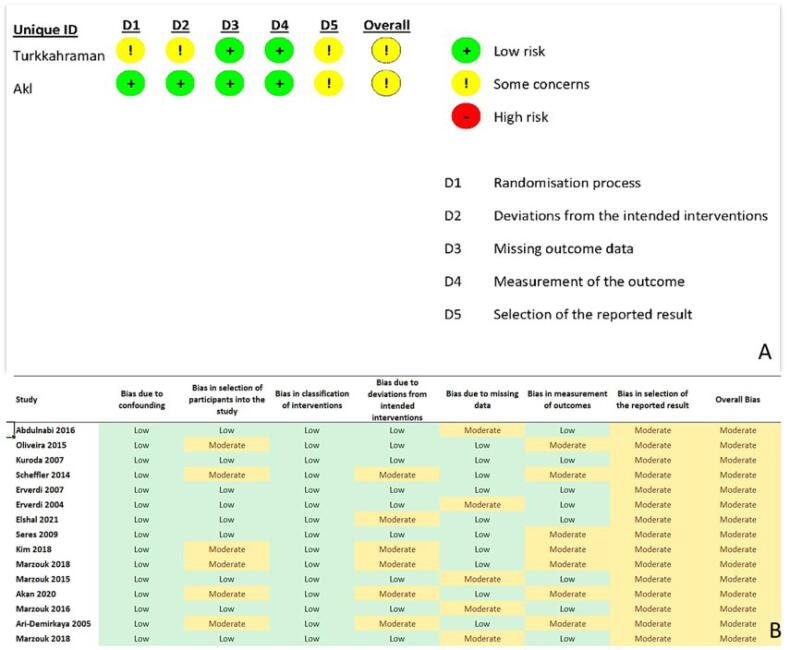
Risk of bias summary. a: RoB2, b: ROBINS-I

 For non-randomized studies, the ROBINS-I tool was used to assess the quality of studies. In this section, all available studies were classified as moderate regarding the overall risk of bias. The outcomes of these assessments are presented in Figures 2a and 2b, respectively.

###  Synthesis of results

 A random-effects meta-analysis was conducted to evaluate the extent of posterior intrusion during treatment using the U6-PP parameter. Various outcomes were assessed, including dental characteristics (overbite, overjet), cephalometric skeletal measurements (SNA, SNB, ANB, S-N-Pog, mandibular plane angle, palatal plane angle, LAFH), cephalometric dental measurements (occlusal plane angle, U1-FH (angle), L1-MP (distance), L6-MP (distance), U1-PP (distance), U6-PP (distance)), and soft tissue evaluations (upper lip to E-line, lower lip to E-line).

###  Meta-analysis for intrusion

**Figure 3:**
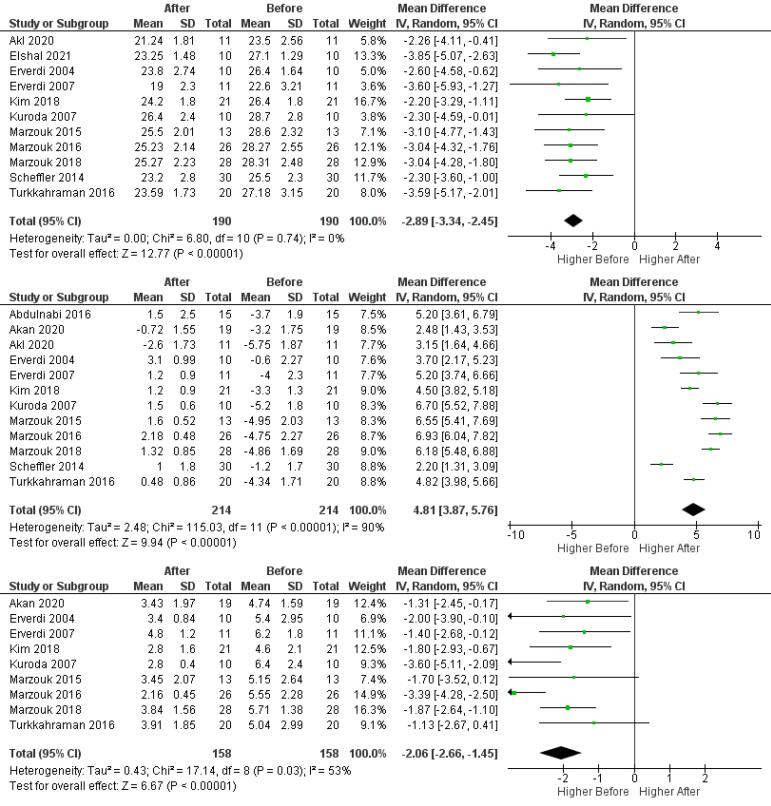
Forrest plot of random-effects analysis of differences of a: U6-pp, b: overbite, and c: overjet

 For the meta-analysis of posterior intrusion (U6-PP), 11 studies and 190 patients were analyzed. The maxillary molars were intruded by an average of 2.89 mm using TADs, which is statistically significant (MD = -2.89, 95% CI: -3.34, -2.45, *P* < 0.00001). Notably, there was minimal heterogeneity among the study results (I^2^=0%), indicating consistency in the findings. The results of this analysis are depicted in Figure 3a.

 Subgroup analysis examined the impact of anchorage type (mini-plates or mini-screws) and force level (150‒250 g or 400‒450 g) on intrusion. Posterior intrusion using mini-plates measured 3.29 mm (MD = -3.29, 95% CI: -3.85, -2.72, *P* < 0.00001), while mini-screws achieved 2.25 mm (MD = -2.25, 95% CI: -2.97, -1.53, *P* < 0.00001). Mini-plates were significantly more effective for posterior intrusion (*P* = 0.03). The results of this analysis are depicted in Figure S1.

 The force level did not significantly affect intrusion, with 150‒250 g yielding 2.83 mm (MD = -2.83, 95% CI: -3.47, -2.20, *P* < 0.00001) and 400‒450 g achieving 2.99 mm (MD = -2.99, 95% CI: -3.68, -2.30, *P* < 0.00001; *P* = 0.74). The results of this analysis are depicted in Figure S2.

###  Meta-analysis for dental characteristics

 In the meta-analysis of dental characteristics, 12 studies and 214 patients were examined. Overbite increased by an average of 4.81 mm (MD = 4.81, 95% CI: 3.87, 5.76, *P* < 0.00001) after treatment, which is statistically significant. The results of this analysis are depicted in Figure 3b.

 Due to the high heterogeneity (I^2^=90%), subgroup analyses separated mini-plate and mini-screw groups, revealing that the overbite increase was not significantly different between the two (P = 0.40). The results of this analysis are depicted in Figure S3.

 Force magnitude did not significantly affect overbite changes, with 150‒250 g increasing by 4.50 mm (MD = 4.50, 95% CI: 3.30, 5.69, *P* < 0.00001) and 400‒450 g by 5.12 mm (MD = 5.12, 95% CI: 3.66, 6.58, *P* < 0.00001; *P* = 0.52). The results of this analysis are depicted in Figure S4.

 In the meta-analysis of overjet changes following posterior intrusion, 9 studies and 158 patients were evaluated. The average overjet reduction was 2.06 mm (MD = -2.06, 95% CI: -2.66, -1.45, *P* < 0.00001), which is statistically significant. This review had moderate heterogeneity (I^2^=53%). The results of this analysis are depicted in Figure 3c.

###  Meta-analysis for skeletal cephalometric characteristics

**Figure 4:**
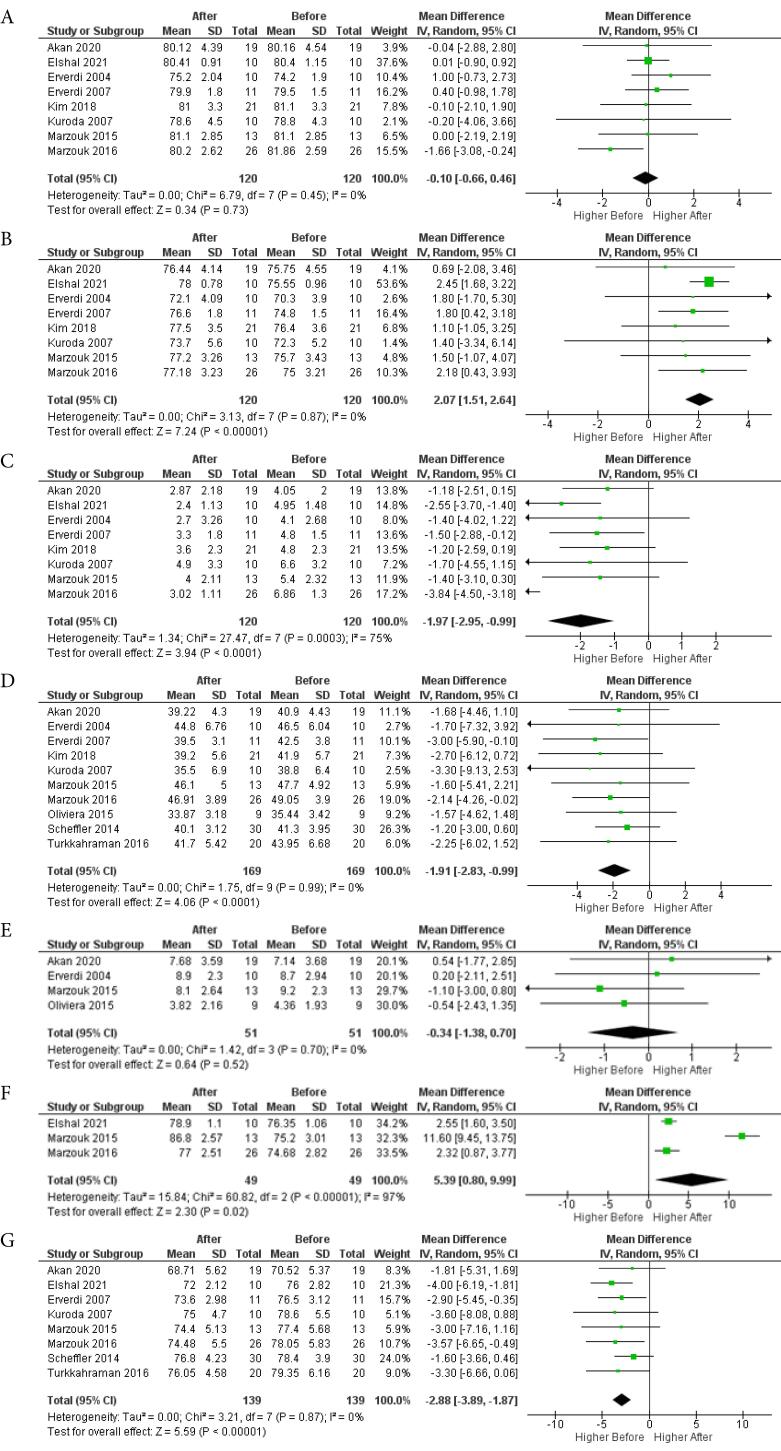
Forrest plot of random-effects analysis of differences in skeletal cephalometric characteristics. a: SNA, b: SNB, c: ANB, d: MP-FH, e: PP-FH, f: SN-Pog, g: LAFH

 For the SNA angle, 8 studies with 120 patients were assessed, resulting in an MD of -0.10 (95% CI: -0.66, -0.46; *P* = 0.73), which was not significant. The SNB angle, with the same patient and study count, had an MD of 2.07 (95% CI: 1.51, 2.64; *P* < 0.00001), while the ANB angle had an MD of -1.97 (95% CI: -2.98, -0.99; *P* < 0.0001). Only the SNA angle change was not significant, with low heterogeneity for SNA and SNB angles (I^2^=0%) and high heterogeneity for ANB angle (I^2^=75%). The results of this analysis are depicted in Figures 4a, 4b, and 4c, respectively.

 For the mandibular plane angle (MP-FH), 10 studies involving 169 patients were analyzed, yielding an MD of -1.91 (95% CI: -2.83, -0.99; *P* < 0.0001), indicating a statistically significant average counterclockwise rotation of 1.91° after posterior intrusion. Heterogeneity was low (I^2^=0%). The results of this analysis are depicted in Figure 4d.

 The palatal plane angle (PP-FH), based on 4 studies with 51 patients, showed an MD of -0.34 (95% CI: -1.38, 0.70; *P* = 0.52), indicating no significant impact on palatal plane rotation, with low heterogeneity (I^2^=0%). The results of this analysis are depicted in Figure 4e.

 For the chin position relative to the anterior cranial base (S-N-Pog), 3 studies with 49 patients revealed an MD of 5.39 (95% CI: 0.80, 9.99; *P* = 0.02), signifying a statistically significant forward movement of the chin by an average of 5.39 mm. However, the high heterogeneity (I^2^=97%) suggests caution in interpreting these results, as subgroup analysis was not possible due to the limited number of studies. The results of this analysis are depicted in Figure 4f.

 Lastly, the meta-analysis for changes in LAFH showed that after posterior intrusion, the lower face was 2.88 mm shorter (MD = 2.88, 95% CI: -3.89, -1.87; *P* < 0.001). This significant result was obtained from 8 studies and 139 patients, with consistent findings (I^2^=0%). The results of this analysis are depicted in Figure 4g.

###  Meta-analysis for dental and soft tissue cephalometric characteristics

**Figure 5:**
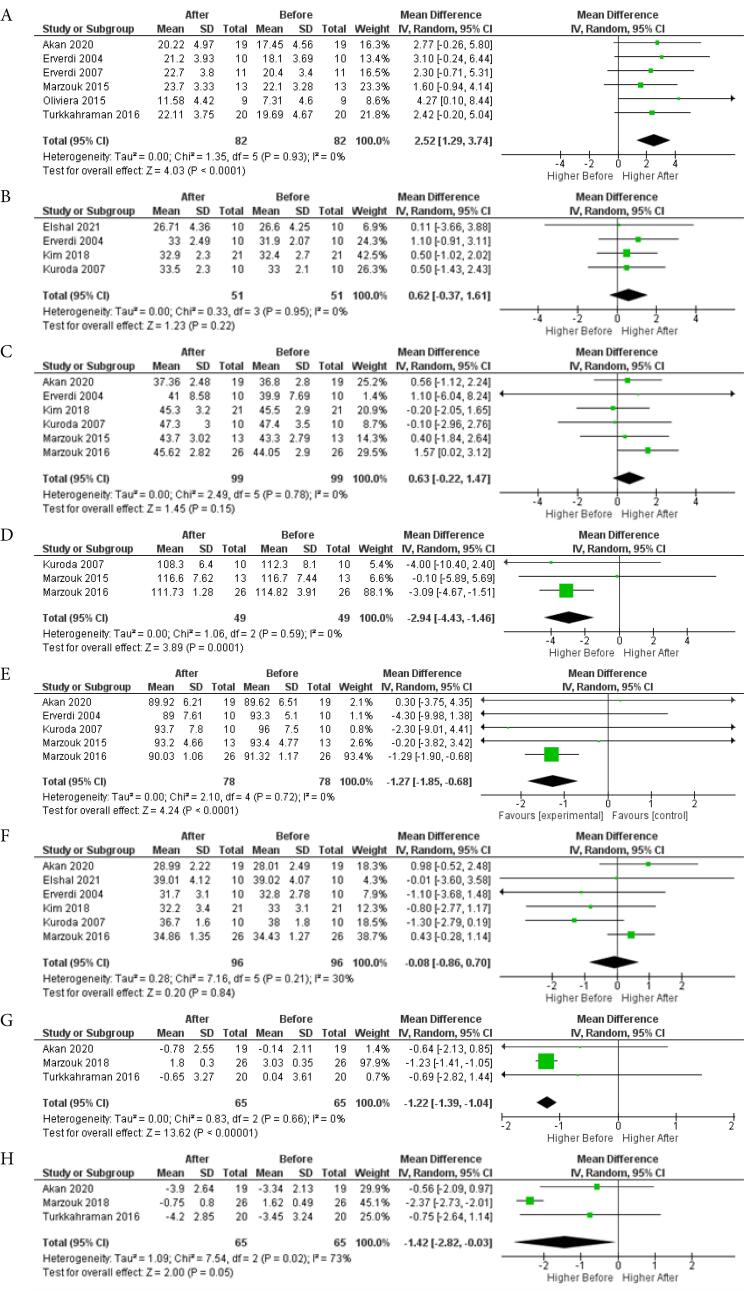
Forrest plot of random-effects analysis of differences in dental and soft tissue cephalometric characteristics. a: OP-FH, b: U1-PP (distance), c: L1-MP (distance), d: U1-FH, e: IMPA, f: L6-MP, g: LL-E line, h: UL-E line

 The dental cephalometric characteristics meta-analysis revealed significant steepening of the occlusal plane (OP-FH) after posterior intrusion, based on 6 studies and 82 patients (MD = 2.52, 95% CI: 1.29, 3.74, *P* < 0.0001), with low heterogeneity (I^2^=0%). The results of this analysis are depicted in Figure 5a.

 Posterior intrusion had no significant effect on the vertical position of upper central incisors (U1-PP) from 4 studies and 51 patients (MD = 0.62, 95% CI: -0.37, 1.61, *P* = 0.22) or lower central incisors (L1-MP) from 6 studies and 99 patients (MD = 0.63, 95% CI: -0.22, 1.47, *P* = 0.15), with low heterogeneity (I^2^=0%). The results of this analysis are depicted in Figures 5b and 5c, respectively.

 The soft tissue characteristics meta-analysis showed that the lower lip moved backward by 1.22 mm relative to the E-line, based on 3 studies and 65 patients (MD = -1.22, 95% CI: -1.39, -1.04, *P* < 0.00001), with low heterogeneity (I^2^=0%). The upper lip also moved backward by 1.42 mm relative to the E-line, based on 3 studies and 65 patients (MD = -1.42, 95% CI: -2.82, -0.03, *P* = 0.05), but with high heterogeneity (I^2^=30%), necessitating cautious interpretation. The results of this analysis are depicted in Figures 5g and 5h, respectively.

 A funnel plot assessing publication bias for posterior intrusion (U6-PP changes) indicated the low potential for publication bias due to asymmetry depicted in Figure S5.

## Discussion

###  Summary of results

 Our meta-analysis showed a significant average intrusion of 2.89 mm for maxillary molars using TADs. Subgroup analyses revealed that miniplates achieved 3.29 mm of intrusion, while miniscrews achieved 2.25 mm of intrusion. The force level did not significantly impact the amount of intrusion. Dental characteristics, such as overbite and overjet, were significantly affected, with overbite increasing by an average of 4.81 mm and overjet decreasing by 2.06 mm. Skeletal cephalometric characteristics showed mixed results, with significant changes in SNB, ANB, mandibular plane angle, chin position, and LAFH, but not in SNA or palatal plane angle. Dental cephalometric characteristics indicated significant steepening of the occlusal plane after posterior intrusion. However, no significant changes in the vertical position of upper and lower central incisors were observed, while maxillary and mandibular incisors were significantly retroclined. The vertical position of the first mandibular molars remained constant during treatment. Soft tissue analysis revealed that both upper and lower lips moved backward relative to the E-line.

###  Factors affecting the outcomes and other considerations

 The advent of TADs like miniplates and miniscrews has markedly transformed orthodontic approaches to open bite malocclusions. Our study demonstrated a significant difference in the amount of molar intrusion achievable via miniplates and miniscrews, with miniplates presenting superior results. The variations stem from factors such as the robustness of anchorage stability, controllability of force application, and potential synergistic impact of adjunctive appliances like posterior bite plates, often coupled with miniplates.

 Interestingly, Turkkahraman et al,^16^ Akl et al,^18^ and Marzouk et al^17^ found divergent impacts on dental parameters, like intermolar distances and molar torques, based on the device employed. Miniplates retained their original molar positions effectively, while mini-screws increased intermolar distances. These distinctions could arise from the interaction of miniplates with occlusal plates, which help maintain intermolar spacing.

 Despite these differences, both devices induce less than 2º of distal tipping in molars; however, miniplates create a more pronounced effect. This outcome is likely due to force application points distal to the molar centers of resistance, inciting a rotational movement. The tipping with miniplates suggests a more distal force application point, which might be attributable to miniplate design or positioning.

 However, the effectiveness of miniplates comes with trade-offs. Although superior in performance and inferior in side effects, miniplates require more specialist appointments, extending treatment timelines and escalating patient expenses. Insertion procedures are more invasive, leading to higher postoperative discomfort and longer recovery periods.

 Post-insertion pain levels are significant considerations. Sreenivasagan et al^27^ and Kawaguchi et al^28^ reported elevated pain levels after miniplate placement, often requiring extended analgesic prescriptions. This discomfort, potentially amplified in cases of poor oral hygiene and resultant inflammation, could negatively influence patient compliance and satisfaction.

 Patient age may affect device selection. According to Chen et al,^29^ mini-screws are recommended for patients aged ≥ 12‒13 due to sufficient bone quality and quantity, while miniplates, less dependent on bone quality, could be used as early as 10 years of age. However, our systematic review was comprised mainly of patients > 15 years old; thus, exploring these devices’ efficacy in younger populations warrants further research.

 Maxillary zygomatic screws provide an intriguing compromise between mini-screws and miniplates. Directly inserted by the treating orthodontist, these screws do not require additional specialist appointments or invasive surgical flap elevation. However, the practitioner must exercise caution due to their proximity to vital structures like dental roots and maxillary sinus.

 Orthodontic treatments’ applied force levels did not significantly impact intrusion amounts, underscoring the need for moderate, controlled force within an optimal range. Excessive force could risk root resorption and damage to periodontal structures.

 Indeed, root resorption was observed in patients undergoing posterior impaction using both mini-screws and miniplates. The clinical significance of root resorption highlights the necessity for vigilant patient selection, strategic treatment planning, and close monitoring throughout treatment.

 Treatment outcome stability is crucial, especially for open bite cases treated with TADs. As shown by Marzouk et al,^21^ relapse rates within the first year reached 10.20% for intruded maxillary molars and 8.19% for overbite, with rates increasing by the fourth year. The correlation of relapse with pretreatment maxillary molar height and open bite severity underscores the need for effective long-term retention strategies.

 Our meta-analysis yielded noteworthy alterations in dental parameters, particularly valuable for open bite patients. It primarily signaled a marked reduction in anterior facial height, demonstrating successful posterior tooth intrusion. Complementing this, an approximately 4-mm shrinkage in the interlabial gap was observed, as per individual data from studies by Marzouk et al^17^ and Akan et al.^20^ This significant transformation indicates an enhanced lip seal, a vital factor for ensuring long-term stability of orthodontic treatment.

 Changes were not confined to hard tissue parameters; soft tissue profile alterations also arose. The mentolabial sulcus angle increased while the facial convexity—assessing facial curvature from the profile view—marginally decreased. These changes stem from the mandible’s autorotation following the intrusion of posterior teeth. This rotational movement can enhance the facial profile, reduce lower lip protrusion, and ultimately boost the overall esthetic treatment outcome—a critical aspect for many patients.

 Improvements in overbite and reductions in overjet were also noticeable. The former signifies the efficacy of impaction techniques utilizing miniplates and mini-screws. At the same time, the latter improvement arises from the autorotation of the mandible and minor changes (approximately 2º) in the mandibular plane rotation. These factors facilitate improved alignment of dental arches, leading to a more stable, functional occlusion and, ultimately, better patient satisfaction.

 Individual study evaluations also showed an increase in the interincisal angle, a trend seen in studies by Kuroda et al,^9^ Erverdi et al,^12^ Marzouk et al,^19^ and Akan et al.^20^ This, along with a decrease in upper and lower incisor angles, suggests a shift of upper and lower incisors towards a more upright position. Such a change enhances the bite and esthetic appearance—a boon for open bite patients who often exhibit a reduced interincisal angle due to forward-inclined incisors.

 During the retention phase, as studied by Kang et al,^30^ a minor increase of 0.92 mm was observed in the vertical distance between the maxillary teeth and palatal plane. Moreover, a review by González Espinosa et al^31^ reported relapse rates around 12% for maxillary molars and 27.2% for mandibular molars, signifying that the stability of open bite correction via molar intrusion using TADs is somewhat comparable to surgical approaches. These insights accentuate the need for treatment planning to consider potential relapse and the importance of effective long-term retention strategies to sustain the treatment outcomes.

 The effects of posterior intrusion on periodontal tissues are crucial as they directly influence oral health. In a study by Ghanbari et al,^32^ significant increases in plaque index scores during treatment indicated heightened plaque accumulation. Similarly, probing pocket depth scores increased significantly over time. However, the average distance from the mini-screw to the gingival level was statistically consistent, except for a minor decrease between baseline and the fifth treatment month. Indices for keratinized gingiva and bleeding on probing remained statistically insignificant.

 Bayani and collegues’ study^33^ found a slight increase in probing depth during active treatment, which was not statistically significant. Interestingly, the gingival margin shifted coronally by an average of 1 ± 0.8 mm, remaining stable during retention, indicating an overall coronal displacement. The study also reported a gain in attachment level, a positive indicator of periodontal health improvement. Despite some alveolar bone resorption during active treatment, most was regained during retention.

 Orthodontic treatment inherently carries risks to root structure, especially when mini-screws or mini-implants are involved. However, this meta-analysis shows only minimal and clinically insignificant root resorption, a promising indicator for molar intrusion techniques. These findings, while encouraging, highlight the necessity for regular radiographic examinations to detect and manage any potential root damage during treatment.

 Furthermore, the interradicular space and proximity to anatomical structures are critical factors when placing TADs. Appropriate placement ensures minimal discomfort and reduces the risk of root damage or impingement on critical anatomical features such as the maxillary sinus or mandibular canal. Careful planning and execution are, therefore, paramount.

 Regarding patient discomfort, most patients reported minimal to moderate pain or discomfort during the first few days following TAD placement, gradually reducing over time. Using appropriate pain management strategies such as analgesics and clear communication about what to expect can enhance patient compliance.

 While this meta-analysis provides a comprehensive look at molar intrusion’s impact on dental and facial characteristics, it also underscores the importance of personalized treatment planning. Factors like the patient’s age, degree of malocclusion, general health status, and patient’s expectations should be thoroughly considered while formulating a treatment strategy. In addition, factors such as potential relapse, periodontal health, root safety, and patient comfort are equally crucial to consider.

## Strengths and Limitations

 The strengths of our study include its comprehensive review of literature on TAD use in open bite treatment, considering various types, and analyzing diverse parameters like dental and facial changes, root resorption, pain levels, and costs. We also included long-term results to understand treatment stability.

 However, limitations exist due to variations in methodologies, sample sizes, and treatment protocols of the reviewed studies. The majority of these studies were observational, implying a higher risk of bias than randomized controlled trials, and measurement inconsistencies across studies might affect data comparison.

 For future research, we suggest focusing on longitudinal and randomized controlled trials to assess long-term stability and compare the effects of different TADs. Additionally, more comparative studies are needed to evaluate the efficacy, safety, impact on patient comfort, cost-effectiveness, and appointment needs of various TADs.

## Conclusion

 In conclusion, our study demonstrated that TADs, such as miniplates and miniscrews, are effective in achieving significant intrusion of maxillary molars, leading to improvements in dental and skeletal characteristics in patients with open bite malocclusion. The maxillary molars were intruded 2.89 mm, and overbite increased by 4.81 mm on average. Miniplates were found to be more effective in achieving greater intrusion compared to miniscrews. However, they also have a higher cost, longer recovery period, cause more discomfort, and may require additional appointments with surgical specialists. The choice of TAD should be based on careful consideration of factors such as patient age, dental and skeletal characteristics, and patient preferences.

 Although our study found that the force level did not significantly impact the amount of intrusion, it is essential to apply controlled and appropriate forces to achieve successful treatment outcomes while minimizing the risk of complications such as root resorption and periodontal issues. Additionally, the long-term stability of treatment outcomes is an important aspect to consider, and effective retention strategies should be implemented to maintain the achieved outcomes.

## Acknowledgments

 This study was extracted from a post-graduate thesis project. Herby, we extend our gratitude to the Research Counselor of Mashhad University of Medical Sciences for the financial support of this research project.

## Competing Interests

 The authors declare no competing interests.

## Ethical Approval

 The study protocol was approved by the Research Ethics Committee of the Mashhad University of Medical Sciences (IR.MUMS.DENTISTRY.REC.1401.41).

## Funding

 This study was supported by the Research Counselor of Mashhad University of Medical Sciences (grant number: 4010136).

## Supplementary Files

Supplementary file 1 contains Figures S1-S5.
