# Supplementary material for: Effects of posterior intrusion using skeletal anchorage on treating anterior open bite: A systematic review and meta-analysis
Source: J Dent Res Dent Clin Dent Prospects. 2023 Dec 30;17(4):196–210. doi: 10.34172/joddd.2023.40754 (PMC10998167; doi:10.34172/joddd.2023.40754)
Supplement: Supplementary file 1 — contains Figures S1-S5. [file joddd-17-196-s001.pdf]

Omidkhoda et al, *J Dent Res Dent Clin Dent Prospects,* 2023, 17(4), 196-S1.
doi: 10.34172/joddd.2023.40754
https://joddd.tbzmed.ac.ir

Supplementary file 1

Study or Subgroup	After Mean	After SD	After Total	Before Mean	Before SD	Before Total	Weight	Mean Difference IV, Random, 95% CI
**4.2.1 Miniplate**								
Elshal 2021	23.25	1.48	10	27.1	1.29	10	13.3%	-3.85 [-5.07, -2.63]
Erverdi 2004	23.8	2.74	10	26.4	1.64	10	5.0%	-2.60 [-4.58, -0.62]
Erverdi 2007	19	2.3	11	22.6	3.21	11	3.6%	-3.60 [-5.93, -1.27]
Marzouk 2015	25.5	2.01	13	28.6	2.32	13	7.1%	-3.10 [-4.77, -1.43]
Marzouk 2016	25.23	2.14	26	28.27	2.55	26	12.1%	-3.04 [-4.32, -1.76]
Marzouk 2018	25.27	2.23	28	28.31	2.48	28	12.9%	-3.04 [-4.28, -1.80]
Turkkahraman 2016	23.59	1.73	20	27.18	3.15	20	8.0%	-3.59 [-5.17, -2.01]
**Subtotal (95% CI)**			**118**			**118**	**62.1%**	**-3.29 [-3.85, -2.72]**
Heterogeneity: Tau² = 0.00; Chi² = 1.84, df = 6 (P = 0.93); I² = 0%								
Test for overall effect: Z = 11.43 (P < 0.00001)								
**4.2.2 Miniscrew**								
Akl 2020	21.24	1.81	11	23.5	2.56	11	5.8%	-2.26 [-4.11, -0.41]
Kim 2018	24.2	1.8	21	26.4	1.8	21	16.7%	-2.20 [-3.29, -1.11]
Kuroda 2007	26.4	2.4	10	28.7	2.8	10	3.8%	-2.30 [-4.59, -0.01]
Scheffler 2014	23.2	2.8	30	25.5	2.3	30	11.7%	-2.30 [-3.60, -1.00]
**Subtotal (95% CI)**			**72**			**72**	**37.9%**	**-2.25 [-2.97, -1.53]**
Heterogeneity: Tau² = 0.00; Chi² = 0.02, df = 3 (P = 1.00); I² = 0%								
Test for overall effect: Z = 6.11 (P < 0.00001)								
**Total (95% CI)**			**190**			**190**	**100.0%**	**-2.89 [-3.34, -2.45]**
Heterogeneity: Tau² = 0.00; Chi² = 6.80, df = 10 (P = 0.74); I² = 0%								
Test for overall effect: Z = 12.77 (P < 0.00001)								
Test for subgroup differences: Chi² = 4.94, df = 1 (P = 0.03), I² = 79.8%								

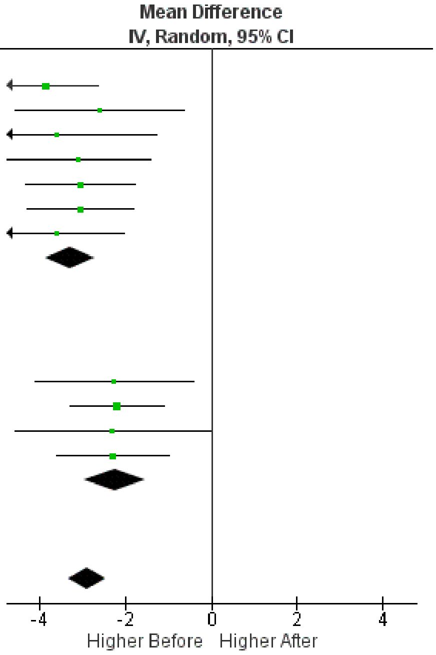

Figure S1: Forrest plot of subgroup analysis of differences in U6-PP according to the type of anchorage used.

Study or Subgroup	Experimental Mean	Experimental SD	Experimental Total	Control Mean	Control SD	Control Total	Weight	Mean Difference IV, Random, 95% CI
**5.2.1 150-250gr**								
Elshal 2021	23.25	1.48	10	27.1	1.29	10	13.3%	-3.85 [-5.07, -2.63]
Erverdi 2004	23.8	2.74	10	26.4	1.64	10	5.0%	-2.60 [-4.58, -0.62]
Kim 2018	24.2	1.8	21	26.4	1.8	21	16.7%	-2.20 [-3.29, -1.11]
Kuroda 2007	26.4	2.4	10	28.7	2.8	10	3.8%	-2.30 [-4.59, -0.01]
Scheffler 2014	23.2	2.8	30	25.5	2.3	30	11.7%	-2.30 [-3.60, -1.00]
Turkkahraman 2016	23.59	1.73	20	27.18	3.15	20	8.0%	-3.59 [-5.17, -2.01]
**Subtotal (95% CI)**			**101**			**101**	**58.5%**	**-2.83 [-3.47, -2.20]**
Heterogeneity: Tau² = 0.09; Chi² = 5.78, df = 5 (P = 0.33); I² = 13%								
Test for overall effect: Z = 8.74 (P < 0.00001)								
**5.2.2 400-450gr**								
Akl 2020	21.24	1.81	11	23.5	2.56	11	5.8%	-2.26 [-4.11, -0.41]
Erverdi 2007	19	2.3	11	22.6	3.21	11	3.6%	-3.60 [-5.93, -1.27]
Marzouk 2015	25.5	2.01	13	28.6	2.32	13	7.1%	-3.10 [-4.77, -1.43]
Marzouk 2016	25.23	2.14	26	28.27	2.55	26	12.1%	-3.04 [-4.32, -1.76]
Marzouk 2018	25.27	2.23	28	28.31	2.48	28	12.9%	-3.04 [-4.28, -1.80]
**Subtotal (95% CI)**			**89**			**89**	**41.5%**	**-2.99 [-3.68, -2.30]**
Heterogeneity: Tau² = 0.00; Chi² = 0.89, df = 4 (P = 0.93); I² = 0%								
Test for overall effect: Z = 8.50 (P < 0.00001)								
**Total (95% CI)**			**190**			**190**	**100.0%**	**-2.89 [-3.34, -2.45]**
Heterogeneity: Tau² = 0.00; Chi² = 6.80, df = 10 (P = 0.74); I² = 0%								
Test for overall effect: Z = 12.77 (P < 0.00001)								
Test for subgroup differences: Chi² = 0.11, df = 1 (P = 0.74), I² = 0%								

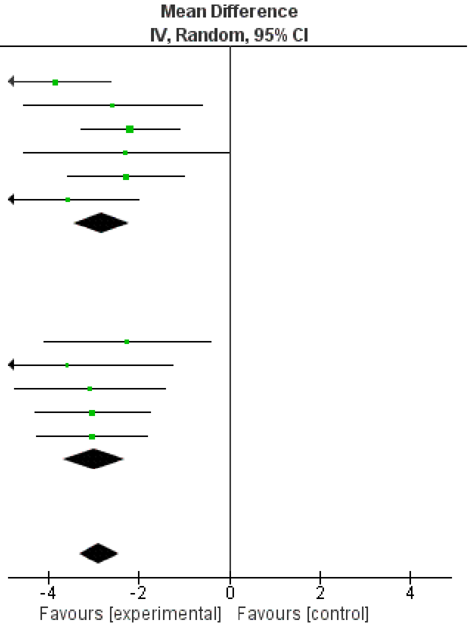

Figure S2: Forrest plot of subgroup analysis of differences in U6-PP according to the amount of applied force.

Study or Subgroup	After Mean	After SD	After Total	Before Mean	Before SD	Before Total	Weight	Mean Difference IV, Random, 95% CI
**4.1.1 Miniplate**								
Akan 2020	-0.72	1.55	19	-3.2	1.75	19	8.5%	2.48 [1.43, 3.53]
Erverdi 2004	3.1	0.99	10	-0.6	2.27	10	7.6%	3.70 [2.17, 5.23]
Erverdi 2007	1.2	0.9	11	-4	2.3	11	7.7%	5.20 [3.74, 6.66]
Marzouk 2015	1.6	0.52	13	-4.95	2.03	13	8.3%	6.55 [5.41, 7.69]
Marzouk 2016	2.18	0.48	26	-4.75	2.27	26	8.7%	6.93 [6.04, 7.82]
Marzouk 2018	1.32	0.85	28	-4.86	1.69	28	9.0%	6.18 [5.48, 6.88]
Turkkahraman 2016	0.48	0.86	20	-4.34	1.71	20	8.8%	4.82 [3.98, 5.66]
**Subtotal (95% CI)**			**127**			**127**	**58.8%**	**5.16 [3.99, 6.33]**
Heterogeneity: Tau² = 2.19; Chi² = 56.24, df = 6 (P < 0.00001); I² = 89%								
Test for overall effect: Z = 8.62 (P < 0.00001)								
**4.1.2 Miniscrew**								
Abdulnabi 2016	1.5	2.5	15	-3.7	1.9	15	7.5%	5.20 [3.61, 6.79]
Akl 2020	-2.6	1.73	11	-5.75	1.87	11	7.7%	3.15 [1.64, 4.66]
Kim 2018	1.2	0.9	21	-3.3	1.3	21	9.0%	4.50 [3.82, 5.18]
Kuroda 2007	1.5	0.6	10	-5.2	1.8	10	8.3%	6.70 [5.52, 7.88]
Scheffler 2014	1	1.8	30	-1.2	1.7	30	8.8%	2.20 [1.31, 3.09]
**Subtotal (95% CI)**			**87**			**87**	**41.2%**	**4.33 [2.79, 5.87]**
Heterogeneity: Tau² = 2.70; Chi² = 41.07, df = 4 (P < 0.00001); I² = 90%								
Test for overall effect: Z = 5.52 (P < 0.00001)								
**Total (95% CI)**			**214**			**214**	**100.0%**	**4.81 [3.87, 5.76]**
Heterogeneity: Tau² = 2.48; Chi² = 115.03, df = 11 (P < 0.00001); I² = 90%								
Test for overall effect: Z = 9.94 (P < 0.00001)								
Test for subgroup differences: Chi² = 0.70, df = 1 (P = 0.40), I² = 0%								

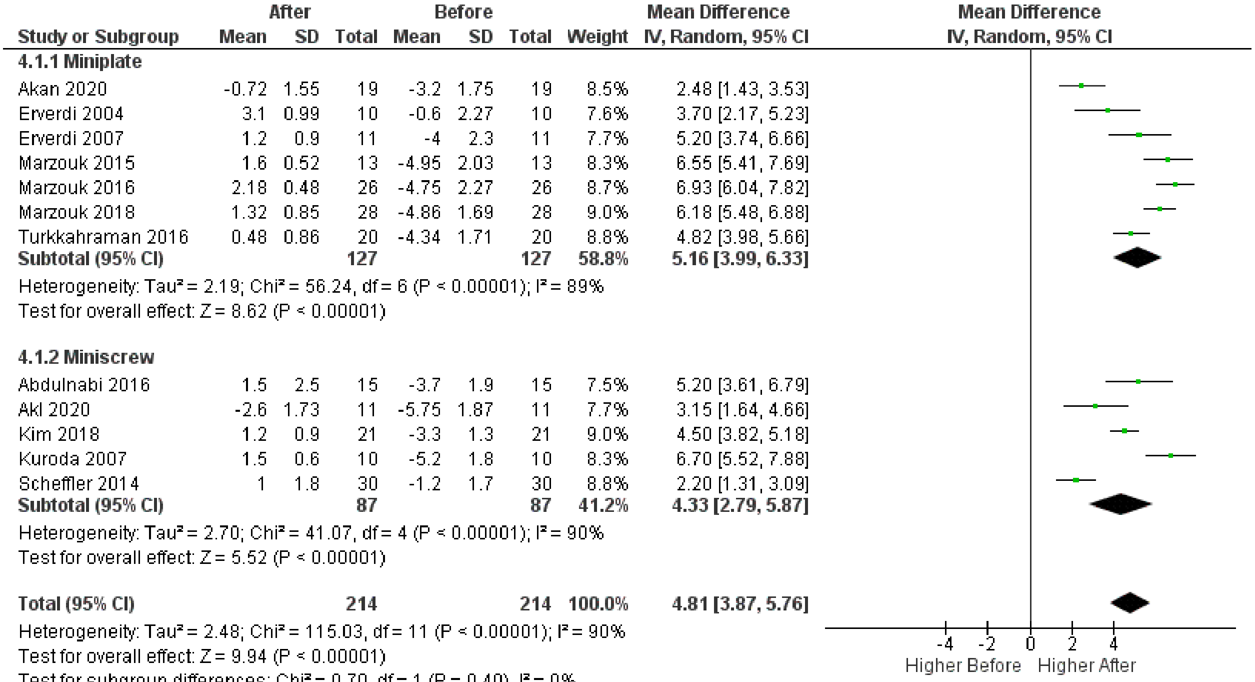

Figure S3: Forrest plot of subgroup analysis of differences of overbite according to the type of anchorage used.

Study or Subgroup	Experimental Mean	Experimental SD	Experimental Total	Control Mean	Control SD	Control Total	Weight	Mean Difference IV, Random, 95% CI
**5.1.1 150-250gr**								
Abdulnabi 2016	1.5	2.5	15	-3.7	1.9	15	7.5%	5.20 [3.61, 6.79]
Erverdi 2004	3.1	0.99	10	-0.6	2.27	10	7.6%	3.70 [2.17, 5.23]
Kim 2018	1.2	0.9	21	-3.3	1.3	21	9.0%	4.50 [3.82, 5.18]
Kuroda 2007	1.5	0.6	10	-5.2	1.8	10	8.3%	6.70 [5.52, 7.88]
Scheffler 2014	1	1.8	30	-1.2	1.7	30	8.8%	2.20 [1.31, 3.09]
Turkkahraman 2016	0.48	0.86	20	-4.34	1.71	20	8.8%	4.82 [3.98, 5.66]
**Subtotal (95% CI)**			**106**			**106**	**50.0%**	**4.50 [3.30, 5.69]**
Heterogeneity: Tau² = 1.89; Chi² = 41.15, df = 5 (P < 0.00001); I² = 88%								
Test for overall effect: Z = 7.38 (P < 0.00001)								
**5.1.2 400-450gr**								
Akan 2020	-0.72	1.55	19	-3.2	1.75	19	8.5%	2.48 [1.43, 3.53]
Akl 2020	-2.6	1.73	11	-5.75	1.87	11	7.7%	3.15 [1.64, 4.66]
Erverdi 2007	1.2	0.9	11	-4	2.3	11	7.7%	5.20 [3.74, 6.66]
Marzouk 2015	1.6	0.52	13	-4.95	2.03	13	8.3%	6.55 [5.41, 7.69]
Marzouk 2016	2.18	0.48	26	-4.75	2.27	26	8.7%	6.93 [6.04, 7.82]
Marzouk 2018	1.32	0.85	28	-4.86	1.69	28	9.0%	6.18 [5.48, 6.88]
**Subtotal (95% CI)**			**108**			**108**	**50.0%**	**5.12 [3.66, 6.58]**
Heterogeneity: Tau² = 3.00; Chi² = 57.99, df = 5 (P < 0.00001); I² = 91%								
Test for overall effect: Z = 6.86 (P < 0.00001)								
**Total (95% CI)**			**214**			**214**	**100.0%**	**4.81 [3.87, 5.76]**
Heterogeneity: Tau² = 2.48; Chi² = 115.03, df = 11 (P < 0.00001); I² = 90%								
Test for overall effect: Z = 9.94 (P < 0.00001)								
Test for subgroup differences: Chi² = 0.42, df = 1 (P = 0.52), I² = 0%								

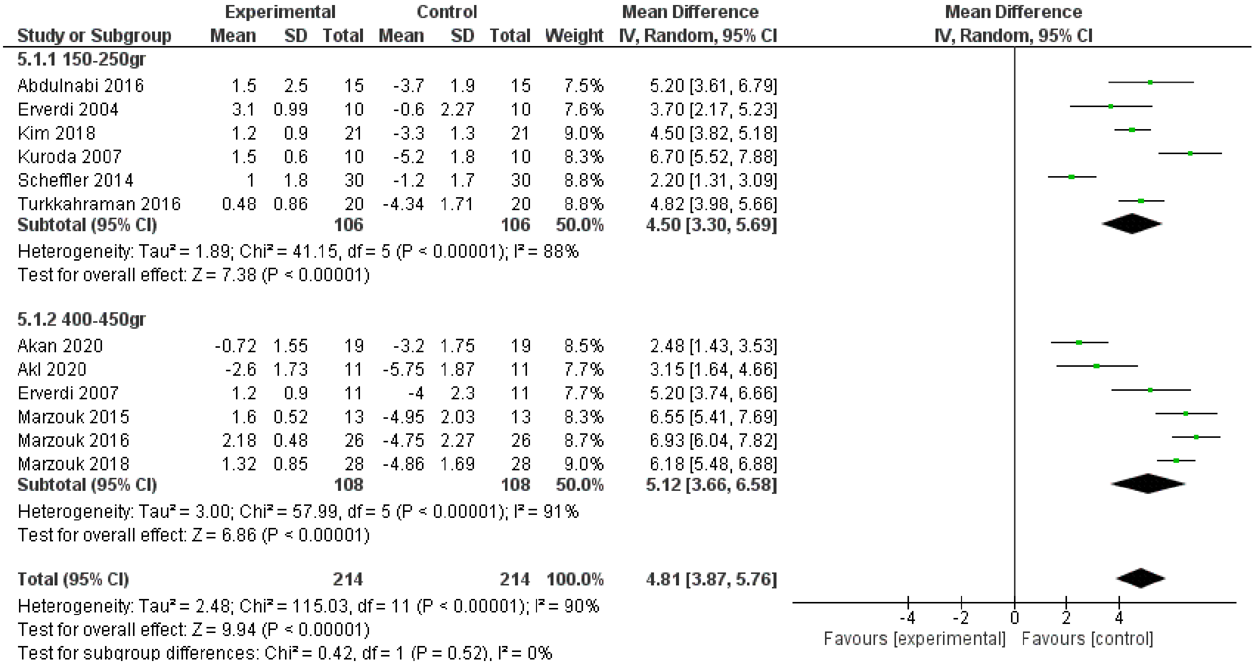

Figure S4: Forrest plot of subgroup analysis of differences of overbite according to the amount of applied force.

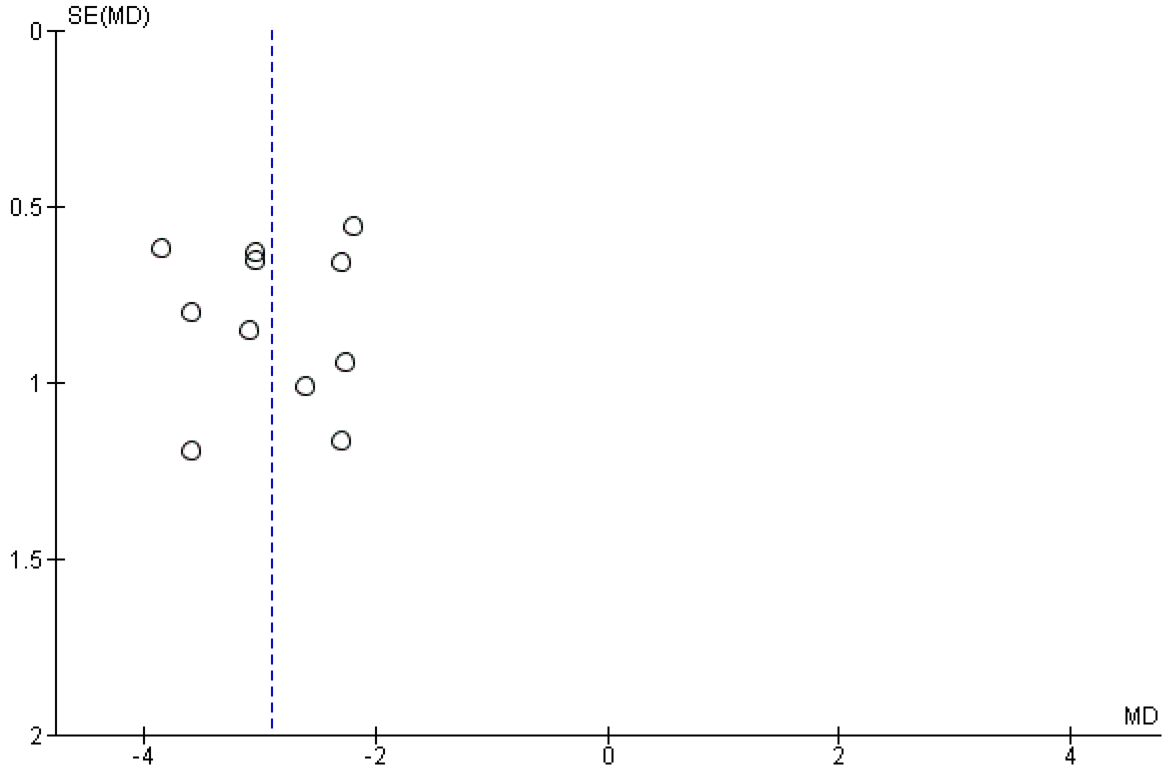

Figure S5: The Funnel plot of U6-PP changes to check publication bias.
